# Safety and efficacy of surgical fixation of fibula fractures using an intramedullary nail: a retrospective observational cohort study in 30 patients

**DOI:** 10.1186/s13037-019-0211-7

**Published:** 2019-10-15

**Authors:** Guilherme Boni, Gustavo T. Sanchez, Gustavo Arliani, Boris A. Zelle, Robinson E. Pires, Fernando B. dos Reis

**Affiliations:** 1 Department of Orthopaedics and Traumatology, Federal University of São Paulo, São Paulo, SP Brazil; 2 IFOR - Instituto de Fraturas, Ortopedia e Reabilitação, São Paulo, SP Brazil; 3 HSC - Hospital São Camilo, São Paulo, SP Brazil; 4 Department of Orthopaedics, University of Texas Health Science Center at San Antonio, 7703 Floyd Curl Dr, MC-7774, San Antonio, TX 78229 USA; 5 Department of the Locomotor Apparatus, Federal University of Minas Gerais, Belo Horizonte, MG Brazil

**Keywords:** Fibula fracture, Intramedullary fixation, Patient safety; quality of life

## Abstract

**Background:**

Open reduction and internal fixation remains the standard treatment for displaced unstable ankle fractures. Plate fixation represents the most frequently used instrumentation option in fibula fractures and favourable outcomes have been reported. Recently, intramedullary nailing techniques have been suggested as a viable alternative resulting in less soft tissue disruption. The objectives of this study are to describe the surgical technique and to evaluate the safety and efficacy of using an intramedullary nail in patients undergoing surgical fixation of their fibula fracture.

**Methods:**

A total of 30 skeletally mature patients with unstable ankle fracture who underwent intramedullary fixation of their fibula fractures from February 2016 to July 2017 were included in this retrospective study. Patients were evaluated using the Short Form-36 (SF-36) and the American Orthopaedic Foot and Ankle Society (AOFAS) at 18 months after surgery.

**Results:**

All patients went on to fracture union. Two patients required a secondary surgical procedure. No patient included in this series developed any wound complications. The mean Physical Component Summary (PCS) of the SF-36 was 53.90 ± 13.3 and the mean Mental Component Summary Score (MCS) was 52.63 ± 11.12. The AOFAS subscale scores were 34.67 ± 1.03 for pain, 42.40 ± 0.2997 for function and 9.50 ± 0.2785 for alignment.

**Conclusions:**

Our study demonstrates promising outcomes associated with intramedullary nail fixation of unstable fibula fractures. We recommend intramedullary nail fixation of fibula fractures to be a safe procedure with a low complication rate.

**Level of evidence:**

Level 4 retrospective case series.

## Background

Ankle fractures account for approximately 9% of all fractures and up to 22% of all lower limb fractures [1, 2]. While many ankle fractures can be treated non-operatively, open reduction and internal fixation remains the standard treatment for unstable and displaced ankle fractures [1]. The primary objectives of surgical treatment are to restore the ankle anatomy and allow for early mobilization. Internal plate fixation remains the most commonly used instrumentation option in the treatment of unstable fibula fractures [3]. Despite favourable outcomes associated with the use of plate fixation, the potential risks of wound dehiscence, infection, and implant failure, continue to represent areas of concern, in particular in patients with diabetes, elderly patients, and patients with significant soft tissue injuries [3, 4]. In addition, hardware prominence remains a common complaint and frequently requires implant removal [5, 6]. In order to address these potential shortcomings of fibula plate fixation, intramedullary nailing has been explored as an alternative treatment option [7]. In particular, patients with significant injuries to the surrounding soft tissue envelope may potentially benefit from this technique [8]. Despite these potential advantages, the safety and efficacy of intramedullary nail fixation of fibula fractures requires further investigation.

The objectives of this study are to describe the surgical technique and to evaluate the safety and efficacy of intramedullary nail fixation of fibula fractures. We hypothesize that intramedullary nail fixation will be associated with a relatively low rate of secondary surgical procedures as compared to traditional fixation methods reported in the literature.

## Methods

This retrospective study was approved by the Institutional Review Board (IRB) of the Federal University of São Paulo (protocol 1,880,524). All patients participating in this study were provided full disclosure and written informed consent was obtained from all subjects enrolled.

Patients with unstable ankle fractures treated February 2016 and July 2017 were included in this retrospective study. The inclusion criteria for enrolment in this study included skeletally mature patients with unstable fibula fractures (Danis-Weber [9] type B and type C) treated with an intramedullary nail. Fracture instability was determined by the treating surgeon, based on incongruency of the mortise, medial clear space widening, syndesmosis widening, or significant fracture displacement. Skeletally immature patients as well as patients, who were unwilling to provide consent, were excluded from this study. All patients were operated on by the same team of board-certified orthopaedic surgeons. In all patients, surgical fixation of the fibula was performed with an intramedullary locked reamed nail (Acumed® solid titanium rod, Hillsboro, OR, United States).

### Surgical technique

**Fig. 1:**
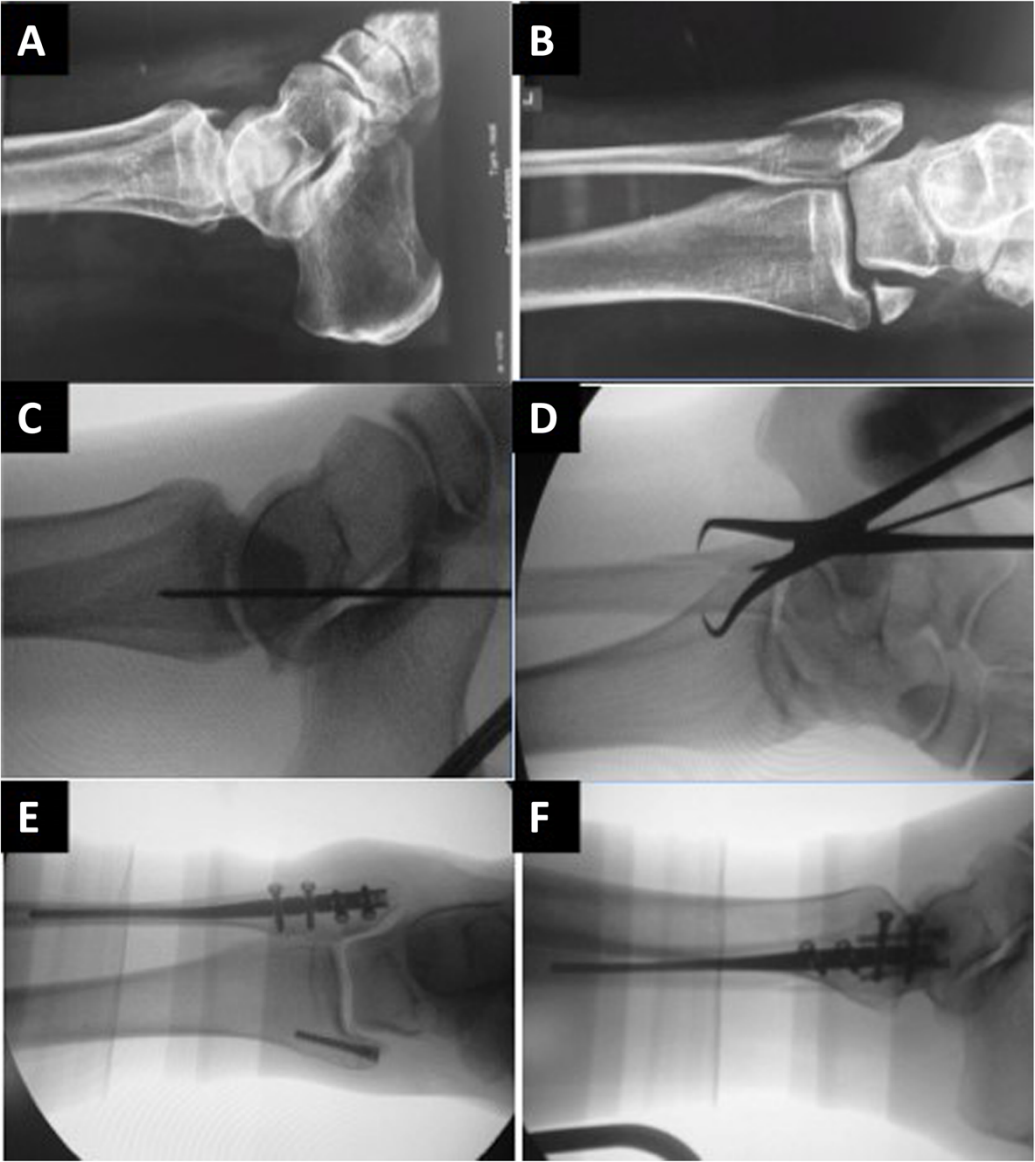
Lateral and anteroposterior radiographs of the ankle in a patient with a displaced bimalleolar ankle fracture (1A-B). Intraoperative fluoroscopic images showing the passage of the guidewire at the tip of the lateral malleolus and reduction with a percutaneous reduction forceps (1C-D). Fluoroscopic images documenting acceptable fracture reduction and appropriate implant position (1E-F)

The surgery is performed in the supine position on a standard radiolucent operating room Table. A minimal-invasive reduction is performed using an approximately 1-cm lateral incision directly over the fracture site in order to allow for direct visualization of the fracture reduction. The fracture reduction is maintained by application of a percutaneously placed pointed reduction clamp. A small stab incision is made over the distal tip of the fibula. The intramedullary canal is accessed using a 6.1-mm cannulated opening reamer, which is placed over a 1.6-mm guidewire. The diaphysis is reamed with a 3.1-mm or 3.7-mm cannulated reamer to prepare the canal for stem placement. The implant is available in two diameters (3 mm and 3.6 mm) and three lengths (110 mm, 145 mm and 180 mm). Associated syndesmotic injuries can be addressed by placement of syndesmotic screws inserted through the nail in a percutaneous fashion. Two anterior to posterior interlocking screws and two lateral to medial interlocking screws are used to lock the nail distal and proximal to the fracture (Fig. 1).

Our postoperative treatment protocol includes toe-touch weight bearing immediately after surgery and initiation of early range of motion exercises. At 4 weeks after surgery, patients are allowed to initiate weight bearing as tolerated to the injured lower extremity. At 2 months after surgery, patients are cleared for all activities of daily living. Patients are allowed to return to sports at 3months postoperatively.

### Collection of outcome data

Clinical and radiographic follow-up data were recorded at subsequent routine follow-up appointments up until 18 months after surgery. Standardized postoperative radiographs including anteroposterior, lateral, and mortise views were obtained during the follow-up visits in order to evaluate fracture healing, nail position, and maintained fracture reduction. The main outcome measure was need for secondary surgical procedures. Secondary outcome measures included surgical site complications, such as non-union, loss of reduction, hardware loosening, wound dehiscence, infections, peroneal tendon injuries, or peri-implant fractures. Fracture union was defined as presence of bridging callus on three out of four cortices on anteroposterior and lateral views. Additional secondary outcome measures included the functional outcomes at 18 months following surgery as measured by the Short Form-36 (SF-36) [10] as well as the American Orthopaedic Foot and Ankle Society (AOFAS) score [11]. The SF-36 is a widely-used standardized scoring system for measuring the health-related quality of life. It has been validated and in various languages and has been used in numerous clinical orthopaedic and non-orthopaedic studies [12]. The SF-36 allows for calculation of eight subscales including physical functioning, role physical, bodily pain, general health, vitality, social functioning, role emotional, mental health. The results can be summarized in the Physical Component Summary (PCS) measure and the Mental Component Summary (MCS) measure. The scores range between 0 and 100 with higher scores reflecting superior quality of life. The AOFAS was used as a foot and ankle specific outcome measure. This clinical rating system includes both patient-reported outcomes as well as provider-reported outcomes based on a physical examination assessing motion, stability, and alignment. The AOFAS can be divided into three subscales pain, function and alignment. The maximum score for each of these subscales is 40 points for pain, 50 points for function, and 10 points for alignment. The AOFAS sum scores range from 0 points (maximum impairment) to 100 points (no impairment). The AOFAS has undergone multiple validation studies and remains one of the most widely-used foot and ankle specific outcome measures [13].

### Statistical analysis

We calculated the the AOFAS and SF-36 score using the standard scoring algorithms for the two questionnaires. The database was maintained in Microsoft Excel 2010 sheets (Microsoft, Redmond, WA). All continuous variables were tested for normal distribution using the Kolmogorov-Smirnov test. We expressed data as means, standard deviations and percentages. We used the used SPSS 22.0 for Mac software in the analysis.

## Results

A total of 30 patients (12 female, 18 male), with unstable fibula fractures were surgically treated using this fixation method. All patients were operated on within 24 h of their injury. The mean age of patients was 50.5 ± 12.61 years. Danis-Weber type B fractures accounted for 70% (*n* = 21) and Danis-Weber type C fracture accounted for 30% (*n* = 9) of all fractures. A total of 17 fractures (56.7%) presented as lateral malleolus fractures and 13 fractures (43.3%) presented as bimalleolar fractures.

### Outcomes

All patients included in this series completed their follow-up and no patients were lost to follow-up. Regarding our main outcome measure, two patients required a secondary surgical procedure. The first patient experienced a significant loss of reduction, because he started playing volleyball at 7 days after surgery, and the radiographs showed a loss of reduction with 10° of valgus malalignment. The second patient required a revision surgery due to a new injury during the postoperative period. This patient suffered a fall of the ladder on the twelfth day after surgery resulting in new peri-implant fracture around the distal portion of the nail. In this patient, the fibula nail was converted to a plate fixation construct and the fibula fracture ultimately healed uneventfully.

Regarding our secondary outcome measures, non-unions, hardware loosening, wound dehiscences, infections, peroneal tendon injuries were not observed in this study. **Two** patients suffered loss of reduction less than 2 mm. This was noticed at the two-months and 3 months follow-up appointments, respectively. As both patients remained asymptomatic, a revision surgery was not deemed to be necessary by the treating surgeon. Overall, patients achieved favourable SF-36 scores with a mean Physical Component Summary (PCS) of 53.90 ± 13.3 and a mean Mental Component Summary Score (MCS) of 52.63 ± 11.12. Table 1 shows the results of the eight SF-36 subscales. The AOFAS subscale scores were 34.67 ± 1.03 for pain, 42.40 ± 0.2997 for function and 9.50 ± 0.2785 for alignment. The results of the AOFAS scales are demonstrated in Table 2.

**Table 1:** SF-36 scores at 18 months following surgery

Domains	Maximum	Minimum	Mean Score	Standard deviation	Mean Normative Data
Physical Function	100	65	79.34	20.03	82.45
Role Physical	100	76	88.34	21.50	74.73
Pain	97	84	82.43	14.89	67.53
General health	95	88	86.56	8.37	71.10
Vitality	100	79	81.66	19.79	66.85
Social aspects	100	72	88.75	19.75	78.30
Role Emotional	94	79	84.40	15.83	70.02
Mental health	100	92	89.99	6.44	73.82

**Table 2:** AOFAS subscales and sum score at 18 months follow-up

AOFAS	Maximum	Minimum	Mean	Standard deviation
Pain	40	20	34.67	1.03
Function	50	34	42.401	0.2997
Alignment	10	8	9.500	0.2785

## Discussion

Unstable ankle fractures are commonly treated by open reduction and internal fixation, using techniques that can be associated with several complications including, loss of reduction, wound healing problems, and infections. In addition, several common symptoms include pain, stiffness, swelling, and instability of the ankle joint [14]. As many as 17–24% of patients may experience unsatisfactory outcomes [15]. The use of an intramedullary fibular nail represents has been suggested as a feasible alternative to plate fixation with the potential to reduce surgical site complications [16]. Our study data suggests that the use of a fibular nail in patients with unstable ankle fractures is associated with a low complication rate and satisfactory clinical outcomes. We suggest that intramedullary nail fixation of unstable ankle fractures represents a safe surgical procedure.

The outcomes achieved in our patient series compare favorably with previously reported studies. Obremskey et al. [17] reported on 20 Weber type B and C ankle fractures treated with plate fixation and recorded an overall SF-36 score of 71.56 at 20 months post-surgery. In an observational study, Bhandari et al. [18] reported on 30 patients with unstable ankle fractures undergoing fibula plate fixation. These authors recorded a mean SF-36 score of 69.78 at 24 months after surgery. Moreover, the average physical function score at 20 and 24 months was 64.7 and 67.5, respectively. In this context, the SF-36 scores recorded in our study suggest very favorable functional outcomes that can be achieved using this fibula fixation technique. We were able to demonstrate a low rate of secondary surgical procedures and high patient satisfaction scores using standardized and validated health-related quality of life scores, which allow for appropriate comparisons with other patient populations [19]. Surprisingly, some of the SF-36 scales in our series trended even higher than in the normative patient population. The safety data recorded in this study are also in line with recent publications reporting relatively low complication rates associated with the use of a locked intramedullary fibular nail [20–23].

Our study has both strengths and limitations. We suggest that a strength of our study is the single center experience with a homogenous patient population and a standardized surgical and rehabilitation protocol. In addition, we were able to record appropriate follow-up information as all patients enrolled in this study completed their follow-up. Limitations of our study include its retrospective design. In addition, we do not have a comparison group from our center and we can only compare our results with data from the literature. In addition, we report on a relatively small sample size and the exact infection rates may require further investigation in larger series and meta-analyses of the available literature. Future studies may further delineate the specific indications for intramedullary nail fixation of the fibula versus other traditional fixation techniques.

## Conclusion

Our results demonstrate the potential of intramedullary nail fixation as a safe method for surgical treatment of unstable fibula fractures. The results of our study suggest promising clinical and functional outcomes as well as low complication rates.

## Data Availability

The datasets generated and/or analyzed during the current study are not publicly available due the presence of personal health information but available from the corresponding author on reasonable request.

## Abbreviations

AOFAS: American Orthopaedic Foot and Ankle Society
MCS: Mental Component Summary
PCS: Physical Component Summary
SF-36: Short Form-36
